# Intrinsic and Synaptic Dynamics Contribute to Adaptation in the Core of the Avian Central Nucleus of the Inferior Colliculus

**DOI:** 10.3389/fncir.2019.00046

**Published:** 2019-07-16

**Authors:** Sebastian T. Malinowski, Jana Wolf, Thomas Kuenzel

**Affiliations:** ^1^Auditory Neurophysiology Group, Department of Chemosensation, RWTH Aachen University, Aachen, Germany; ^2^Department of Chemosensation, RWTH Aachen University, Aachen, Germany

**Keywords:** auditory midbrain, inferior colliculus, avian, response adaptation, *Gallus gallus*, acute brain slice, fiber stimulation

## Abstract

The reduction of neuronal responses to repeated stimulus presentation occurs in many sensory neurons, also in the inferior colliculus of birds. The cellular mechanisms that cause response adaptation are not well described. Adaptation must be explicable by changes in the activity of input neurons, short-term synaptic plasticity of the incoming connections, excitability changes of the neuron under consideration or influences of inhibitory or modulatory network connections. Using whole-cell recordings in acute brain slices of the embryonic chicken brain we wanted to understand the intrinsic and synaptic contributions to adaptation in the core of the central nucleus of the inferior colliculus (ICCc). We described two neuron types in the chicken ICCc based on their action potential firing patterns: Phasic/onset neurons showed strong intrinsic adaptation but recovered more rapidly. Tonic/sustained firing neurons had weaker adaptation but often had additional slow components of recovery from adaptation. Morphological analysis suggested two neuron classes, but no physiological parameter aligned with this classification. Chicken ICCc neurons received mostly mixed AMPA- and NMDA-type glutamatergic synaptic inputs. In the majority of ICCc neurons the input synapses underwent short-term depression. With a simulation of the putative population output activity of the chicken ICCc we showed that the different adaptation profiles of the neuron classes could shift the emphasize of stimulus encoding from transients at long intervals to ongoing parts at short intervals. Thus, we report here that description of biophysical and synaptic properties can help to explain adaptive phenomena in central auditory neurons.

## 1. Introduction

Adaptation, the reduction of neuronal responses to repetitive stimuli, is a common phenomenon throughout several sensory modalities (Hille, 2001), including the auditory system. The reduction of the overall strength of the response of a given neuron to repeated presentation of the same stimulus is called response adaptation (Singheiser et al., 2012b; Pérez-González and Malmierca, 2014). Response adaptation depends on the interval of presentation (Gutfreund and Knudsen, 2006; Singheiser et al., 2012a), persists even in the absence of the stimulus for a certain recovery time and is thus implicated in diverse perceptual phenomena like forward masking (Nelson et al., 2009) or the precedence effect (Litovsky and Yin, 1998; Spitzer et al., 2004). Response adaptation occurs in several auditory brain areas (Ulanovsky et al., 2003; Ingham and McAlpine, 2004; Dean et al., 2005; Wang and Peña, 2013; Ferger et al., 2018) and time-constants of release from adaptation in the hundreds of milliseconds were measured (Ingham and McAlpine, 2004; Gutfreund and Knudsen, 2006; Singheiser et al., 2012a; Wang and Peña, 2013; Ferger et al., 2018). In higher stations of the auditory pathway a related type of adaptation was described: neurons adapt their response to repeated presentation of a certain stimulus but respond normally to a qualitatively different, uncommon stimulus. This phenomenon was termed stimulus-specific adaptation (Ulanovsky et al., 2003; Pérez-González and Malmierca, 2014) and it was described in the mammalian auditory cortex, auditory thalamus and the inferior colliculus (IC) (Malmierca et al., 2009, 2015; Ayala and Malmierca, 2012) and also in the external nucleus of the IC (Reches and Gutfreund, 2008) and forebrain areas (Reches et al., 2010) of birds.

Adaptation is commonly regarded as a type of short-term memory function emerging from properties of the neuronal network (Pérez-González et al., 2012; Ayala et al., 2016). Due to its stimulus-driven properties and the relative conceptual simplicity, a complete understanding of its mechanisms seems possible. This makes adaptation an appealing target for experimental analysis of higher brain function, both on cellular and network levels. Few attempts are however usually made to mechanistically describe adaptive phenomena on the level of the synaptic physiology and the biophysics of the neurons. We want to argue here that any adaptation of the response of a given neuron must be explicable by either upstream changes in input rate, short-term synaptic plasticity of the ascending connections (Friauf et al., 2015), intrinsic biophysical excitability changes of the neuron under consideration (Debanne et al., 2019) or, indeed, influence of the network in the form of lateral or descending inhibitory or modulatory synaptic connections (Ayala et al., 2016). In order to fully understand an adaptative phenomenon it is crucial to take the biophysical and synaptic factors that dynamically shape information processing at a given neuron into account. In order to attempt such a dissection of factors underlying adaptation we recorded with *in-vitro* whole-cell techniques from neurons in the avian core of the central nucleus of the IC (ICCc), which exhibit response adaptation (Singheiser et al., 2012a) *in vivo*. We will describe here the intrinsic dynamics of excitability and the dynamics of ascending lemniscal axonal connections and how these two factors differ for two physiologically defined neurons classes in the avian IC. Since the neurons we analyzed are the starting points of the input stream for space-specific neurons in the external nucleus of the IC, we will also provide *in-silico* experiments to assess the impact of the response dynamics of ICCc neurons on sound processing further along in the auditory pathway. Thus, our study contributes to mechanistic understanding of a higher brain function, adaptation, on the level of cellular physiology and biophysics.

## 2. Materials and Methods

### 2.1. Animal Handling and Brain Slice Preparation

A total of 76 chicken embryos between embryonic day 19 and 21 (HH stages 45/46) were sacrificed for this study. All experimental procedures performed on animals in this study were approved by the local animal welfare officer and state authorities (Landespräsidum für Natur, Umwelt und Verbraucherschutz Nordrhein-Westfalen, Recklinghausen, Germany).

Fertilized chicken eggs (*Gallus gallus domesticus*) were obtained from a local poultry farm (Moonen & Waagemans Kuikenbroeders B.V., Nederweert, The Netherlands) and incubated at 37°C. and 50% relative humidity in a rolling incubator until use. Eggs were then opened and chicken embryos were rapidly decapitated in ovo before any further manipulations. Embryos were staged according to Hamburger and Hamilton (1951).

Standard methods (Weigel and Luksch, 2012) were used to prepare and maintain acute brain slices of embryonic chicken as previously reported (Goyer et al., 2015). Briefly, chicken heads were immediately immersed in ice-cold cutting buffer (im mM: 240 sucrose, 3 KCL, 5 MgCl_2_, 0.5 CaCl_2_, 1.2 NaH_2_PO_4_,23 NaHCO_3_, 11 D-glucose; oxygenated with 95%O_2_/5%CO_2_). Brains were removed from the skull and midbrain hemispheres dissected. Midbrain hemispheres were embedded in agarose gel (2% low melting point agarose, 290 mM saccharose, 2 mM KCL, 3 mM MgCl_2_, 5 mM HEPES). After curing, agarose blocks were trimmed and mindbrains cut into 250 μm slices on a vibratome (Leica VT1200S; Leica Biosystems) while immersed in cutting buffer. Coronal brain slices were cut except for recordings of ascending synaptic connections, which were cut in a parasagittal plane. Slices were transferred for holding into artificial cerebrospinal fluid (ACSF; in mM: 120 NaCl, 3 KCL, 1 MgCl_2_, 2 CaCl_2_, 1.2 NaH_2_PO_4_,23 NaHCO_3_, 11 D-glucose; oxygenated with 95%O_2_/5%CO_2_) and equilibrated at room temperature for 1 h. Immediately prior to transfer into the recording chamber (see next section) the optic tectum overlaying the inferior colliculus were routinely dissected to improve handling of the slices.

### 2.2. Whole-Cell Patch-Clamp Recordings

#### 2.2.1. Recording Setup

Whole-cell patch recordings (Hamill et al., 1981) of neurons in the central nucleus of the inferior colliculus (ICCc) of E19 to E21 embryonic chicken were performed in a custom-built recording setup. The slices were prepared from animals of 19.4 ± 0.6 embryonic days (*N* = 76). Slices containing the IC were transferred into a recording chamber on the stage of an fixed-stage microscope (Nikon Eclipse FN-1) and observed with IR-DIC optics under infrared illumination. The recording chamber was perfused with oxygenated ACSF at room temperature (25°C) at a rate of 100 ml/h (chamber volume was 1.5ml). Neurons in the ICCc were targeted using a 40x immersion objective (Nikon NIR APO 40x/0.80w). ICCc location in frontal and sagittal sections was routinely identified using a histological atlas of the chicken brain (Puelles et al., 2007) and histological experiments prior to this study (data not shown). Patch electrodes were manufactured from borosilicate glass capillaries (GB150F-8P; Science Products, Hofheim, Germany) with a horizontal pipet puller (DMZ Universalpuller; Zeitz Instruments, Martinsried, Germany). For all recordings in this study, pipets were filled with potassium-gluconate based internal solution (in mM: 100 K-gluconate, 40 KCL, 0.1 CaCl_2_, 10 HEPES, 1.1 EGTA, 2 Mg-ATP, 0.4 GTP, 0.1 Alexa Fluor 488 hydrazide, 3mg/ml biocytin; pH adjusted to 7.2 with KOH, osmolarity 280 mOsm; Alexa dye and biocytin obtained from Thermo Fisher Scientific). Liquid junction potential was estimated to be –11 mV and remained uncorrected. Whole-cell recordings were performed with a SEC05LX dSEVC amplifier (npi, Tamm, Germany) in bridge mode or in voltage clamp mode (25 kHz switching frequency). In bridge mode, series resistance compensation was routinely set to twice the electrode resistance measured in the bath solution. This procedure in some cases resulted in residual uncompensated series resistance. Signals were low-pass filtered (5 kHz cutoff frequency) and sampled at 50 kHz (NI-DAQ PCI-6281; National Instruments, Austin TX, USA). Stimulus generation and data collection was performed with custom software written in MATLAB (MATLAB data acquisition toolbox).

#### 2.2.2. Biophysical Characterization

Initial resting membrane potential of all neurons was noted immediately after obtaining whole-cell access. Membrane resistance was measured from sets of small current injections (21 steps from –150 to +300pA) by constructing the current-voltage curves and fitting a linear function to the hyperpolarizing conditions. Exponential functions were fitted to the onset of the voltage responses to estimate the membrane time constant. Membrane time-constant was determined for at least three hyperpolarizing current steps and the values were averaged. Membrane capacitance was then estimated from the measured R_*m*_ and averaged τ_*m*_ readings. Next, action-potential (AP) threshold was determined with repeated single current injections. Then, a current-AP rate relation (F/I) was measured with 10 repetitions of 15 depolarizing current steps between 50 and 300% threshold current. From these F/I curves, AP firing behavior was determined to be either phasic or tonic: phasic neurons fired only one or two AP per stimulus over a wide range of currents while tonic neurons showed an increase of AP numbers with increasing current.

#### 2.2.3. Measurement of Intrinsic Adaptation

Intrinsic adaptation was measured by applying pairs of rectangular stimulus currents (100 ms duration each, amplitude was 300% AP threshold current) with increasing inter-stimulus intervals (1–500 ms in logarithmical steps). For every interval 10 repetitions were recorded, 3 s intervals between repetitions were included to allow recovery of neurons. With the help of custom software AP were automatically detected and analyzed in these recordings. A number of parameters was derived from these data: number of AP per stimulus, latency of the first AP peak re the onset of the stimulus, amplitude of the first AP peak, maximum upward and downward slope of the first AP and finally the width of the first AP measured as the time between the points of steepest up- and downward slope. From these data we constructed adaptation curves by plotting the average “parameter” (from 10 repetitions each) derived from the second stimulus as a function of the inter-stimulus interval, normalized by the average “parameter” derived from the first stimulus. Adaptation curves were then fitted by one of two double exponential functions, depending on the effect of adaptation: if the intrinsic adaptation caused a reduction of the normalized “parameter” (AP count, AP amplitude, AP maximal slopes), we fitted:

(1) $$\begin{array}{l} P\left(ISI\right)=1-\left[\left(A_{fast}\cdot e^{\frac{-ISI}{\tau_{fast}}}\right)+\left(A_{slow}\cdot e^{\frac{-ISI}{\tau_{slow}}}\right)\right] \end{array}$$

with P(ISI) describing the change of the “parameter” as a function of ISI. If intrinsic adaptation caused an increase of the normalized “parameter” (AP latency, AP width), we fitted:

(2) $$\begin{array}{l} P\left(ISI\right)=1+\left[\left(A_{fast}\cdot e^{\frac{-ISI}{\tau_{fast}}}\right)+\left(A_{slow}\cdot e^{\frac{-ISI}{\tau_{slow}}}\right)\right] \end{array}$$

From this double-exponential fits we derived a weighted exponential time-constant:

(3) $$\begin{array}{l} \tau_{w}=\frac{\left(A_{fast}\cdot \tau_{fast}+A_{slow}\cdot \tau_{slow}\right)}{A_{fast}+A_{slow}} \end{array}$$

The weighted time-constants of all cells (grouped according to either AP firing behavior (see above) or dendrite morphology (see below) were averaged and compared. We also averaged the adaptation functions (i.e., the parameter vs. ISI functions per cell) of all cells and fitted Equations 1 or 2 to these average adaptation functions as appropriate.

#### 2.2.4. Measurement of Synaptic Events and Pharmacology

Synaptic events in ICCc neurons were evoked by electrically activating axons ascending into to ICCc. For this we placed a bipolar tungsten electrode (2*MΩ* impedance) in the hilus of the lateral lemniscus fibers entering the IC. This structure is labeled “*HilT*” in the atlas by Puelles et al. (2007). Indeed, parallel ascending fibers were clearly visible in IR-DIC optics in parasagittal slices in the region depicted in the atlas. Stimulation pulses (200 μs duration, monopolar, up to 90 V; mean 42 ± 15*V, n* = 20) were generated with a Grass S44 stimulator and delivered through an AMPI-Flex optical stimulus isolator (A.M.P.I, Jerusalem, Israel). We either recorded post-synaptic potentials in bridge-mode or post-synaptic currents in voltage-clamp mode. For voltage clamp, the cells were held at –60 mV holding potential. We first determined the apparent stimulation threshold by gradually increasing stimulus voltage. All further experiments were then performed at 200% threshold voltage, which may be considered a form of maximum stimulation, although this was not rigorously confirmed for all cells. Blockers for AMPA (6,7-dinitroquinoxaline-2,3-dione, DNQX, 100μM, Tocris/Biotechne) and NMDA (D-2-amino-5-phosphonovalerate, D-AP5, 50 μM, Tocris/Biotechne) type glutamatergic receptors were used for pharmacological dissection. Stimulus protocols for paired-pulse stimulation at various intervals (5–500 ms) were generated by the recording software and repeated 10 times per interval with delays of at least 3s between repetitions to allow the synapses to fully return to steady state. Postsynaptic currents were analyzed with custom software as previously reported (Goyer et al., 2015). Stationary parameters (EPSC amplitude, 10% to 90% risetime, decay time-constant) were measured from the first EPSC of every repetition per cell. Recovery from short-term plasticity was measured by constructing recovery functions from the paired-pulse recordings. Here, the second EPSC amplitude was normalized to the average first EPSC amplitude. Recovery functions were visually categorized into facilitating, depressing or unclear/mixed. Recovery functions of all cells or the respective groups were averaged and fitted with exponential functions. We used a double-exponential function for the average recovery functions of depressing-only or facilitating-only cells:

(4) $$\begin{array}{l} EPSC_{2}\left(ISI\right)=offset+\left[\left(A_{fast}\cdot e^{\frac{-ISI}{\tau_{fast}}}\right)+\left(A_{slow}\cdot e^{\frac{-ISI}{\tau_{slow}}}\right)\right] \end{array}$$

For the average recovery function of all measured cells we used a triple-exponential function to fit a double-exponential depression and an exponential facilitation:

(5) $$\begin{array}{l} EPSC_{2}(ISI)=offset+[\left(A_{fast-depr}\cdot e^{\frac{-ISI}{\tau_{fast-depr}}}\right)\\ \;+(A_{slow-depr}\cdot e^{\frac{-ISI}{\tau_{slow-depr}}})+(A_{facil}\cdot e^{\frac{-ISI}{\tau_{facil}}})] \end{array}$$

Weighted time-constants of the double exponential fits were calculated as in Equation 3 and degree-of-freedom adjusted coefficient of determination (*r*^2^) is given as calculated by the MATLAB function *fit.m*.

### 2.3. Morphological Analysis of Recorded Neurons

Cells were recovered after recording for *post-hoc* anatomical analysis as previously described (Goyer et al., 2016). Briefly, slices were fixed in 4% PFA in phosphate buffer overnight, washed with phosphate buffered saline and 0.3% Triton X-100 multiple times and incubated with streptavidin solution (0.1% Triton X-100, 1% bovine serum albumin in phosphate buffer, 1:800 Alexa-fluor-streptavidin conjugate # S11223 from Thermo Fisher Scientific) for 3h at RT. After multiple washing steps (0.3% Triton X-100 in TRIS-buffered saline solution) a counterstaining with DAPI was performed. Finally, stained slices were mounted in Fluoprep (bioMerieux) between 24 ×60 mm coverslips between frames of 240 μm adhesive sheets (Grace Bio-Labs). Cells were scanned at high magnification with a laser-scanning confocal microscope (Leica TCS SP2, Leica Microsystems) at high z-resolution (≤ 0.5μm per image in confocal stacks). Single images at low magnification were scanned to confirm the location of the cell in the ICCc. 23 cells for which ICCc location could not be confirmed were not included in the adaptation study. For a subset of neurons a full morphological analysis was performed. Soma, axon and dendrites were visually identified and 3D reconstructed using the “Simple Neurite Tracer” plugin (Longair et al., 2011) in FIJI/ImageJ (Schindelin et al., 2012). Reconstructed morphology for soma and dendrites only (omitting the axonal structure, which was only used for visualization of neurons) were analyzed: first, we fitted the minimum volume ellipsoid that enclosed all dendritic points in 3D-space using established algorithms (Aelst and Rousseeuw, 2009) for every cell. We calculated the ratio of the longest and second-longest axis of the resulting ellipsoid to quantify the shape of the dendritic tree. As proposed by Niederleitner and Luksch (2012) neurons with a shape ratio between 1 and 2 were classified as “stellate” and those with a ratio ≥2 were classified as “elongated.” Minimum volume ellipsoids were also fitted to z-projected confocal stacks of non-reconstructed cells in 2D-space. In these cases, the ratio of long to short axis was calculated. Furthermore, we performed a Sholl analysis (Sholl, 1953) in 3D space by counting the number of intersects of dendritic structures with spheres of varying radii centered on the soma for every cell. Number of intersects per sphere was plotted against sphere-radius, data was smoothed by fitting a high order polynomial function. From the Sholl analysis a number of parameters that describe the complexity of the dendritic tree were derived for every cell: the mean value (MV) is the average number of intersects over all radii, the critical value (CV) is the maximum number of intersects, the critical radius (CR) is the radius at which the CV occurs. The maximum distance (MaxDist) is the radius at which no more intersects occur. In addition to these parameters we also extracted the number of branchpoints and the total dendritic pathlength from our 3D reconstruction data. All software for morphological analysis was custom written in Python 2.7.

### 2.4. Data Analysis and Statistics

**Figure 1:**
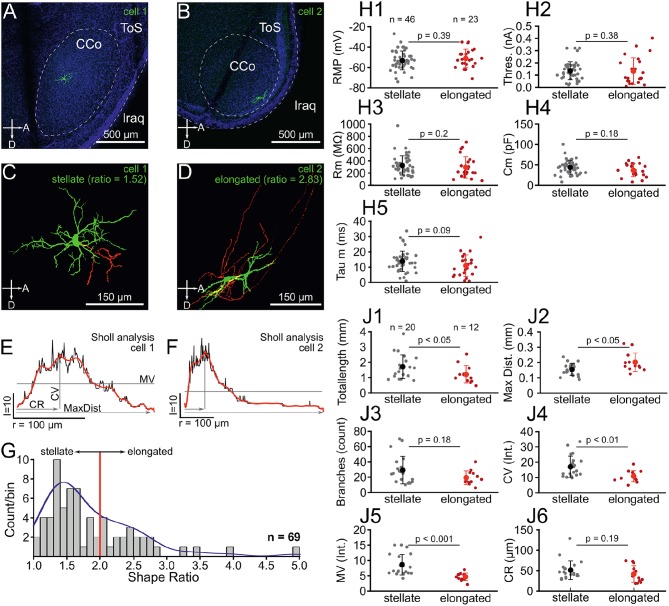
Chick ICCc neurons can be categorized into two classes based on dendritic morphology. **(A,C)** Location **(A)** and 3D-reconstruction **(C)** of an typical ICCc stellate neuron. Abbreviations as in Puelles et al. (2007): ToS, torus semicircularis; CCo, core of the central nucleus; lraq, lateral recess of the aqueduct; A, anterior; D, dorsal. Colors in **(C)** represent: green, soma and dendrites, red, axon. **(B,D)** Location **(B)** and 3D-reconstruction **(D)** of an typical ICCc elongated neuron. **(E)** Sholl-analysis profile of the neuron in **(A,C)** plotted as count of intersects vs. radius of spheres. Depicted are analysis parameters of MV, mean value of intersects; CV, critical value; CR, critical radius; MaxDist, maximal dendritic extent. Black line represents raw data, red superimposed line represents the polynomial fit from which the analysis parameters were extracted. **(F)** Sholl-analysis profile of the neuron in **(B,D)**, presentation as in **(E)**. **(G)** Histogram of dendritic shape ratios. Red line depicts cutoff between stellate and elongated cells (ratio = 2) as defined by Niederleitner and Luksch (2012). Blue line shows probability density function resulting from univariate kernel-density estimate. **(H1–5)** Biophysical parameters of all stellate (gray dots, black marker shows mean ± SEM) and elongated (red dots, red markers shows mean ± SEM) neurons. RMP, resting membrane potential, Thres, action potential threshold current; Rm, membrane input resistance; Cm, membrane capacitance; Tau m, membrane time constant. **(J1–6)** Morphological parameters of dendrites derived from 3D-reconstruction and 3D-Sholl Analysis for all stellate and elongated neurons. Colors and abbreviations: see above.

**Figure 2:**
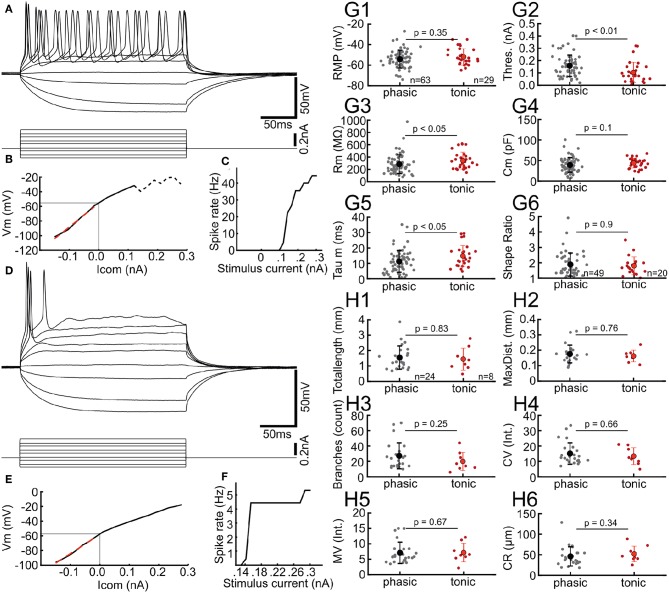
Chick ICCc neurons show two distinct types of AP firing patterns. **(A)** Typical membrane responses to de- and hyperpolarizing current injection of a tonic/sustained neuron. **(B)** Current-Voltage relation of the neuron in (A). Light gray line depicts resting membrane potential. Dashed line depicts conditions where steady-state membrane potential could not be reliable measured due to spiking activity. Red dashed line shows linear fit to small hyperpolarizing current injections. **(C)** Rate of action potential generation (in Hz) upon increasing current amplitudes for the neuron in **(A)**. **(D–F)** Typical membrane responses, current-voltage relation and rate of action potential generation of a phasic/onset neuron. Presentation as in **(A–C)**. **(G1–5)** Biophysical parameters of all phasic (gray dots, black marker shows mean ± SEM) and tonic (red dots, red markers shows mean ± SEM) neurons. Phasic neurons have higher thresholds, lower membrane resistance and lower membrane time-constants. RMP, resting membrane potential, Thres., action potential threshold current; Rm, membrane input resistance; Cm, membrane capacitance; Tau m, membrane time constant. **(G6)** Dendritic shape ratio of all phasic and tonic neurons, colors as in **(G1–5)**. No morphological differences between the groups are visible. **(H1–6)** Morphological parameters of dendrites derived from 3D-reconstruction and 3D-Sholl Analysis for all phasic and tonic neurons. The morphological parameters do not differ between the physiologically defined neurons types. Colors and abbreviations: see above.

**Figure 3:**
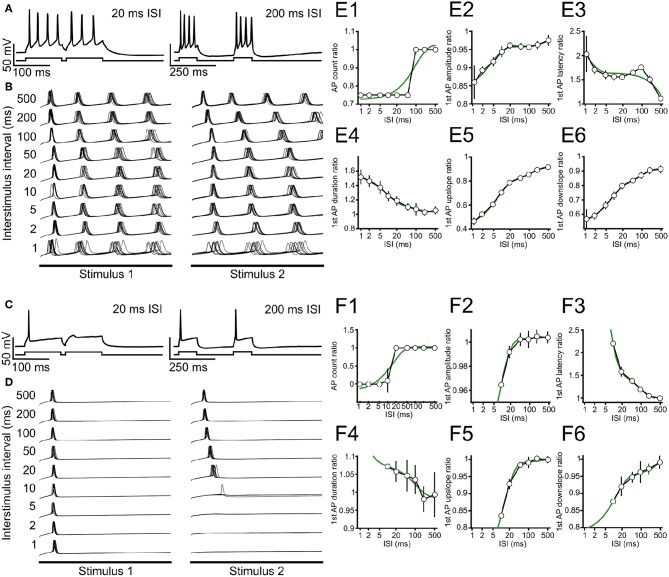
Stimulus-interval dependent adaptation of action potential firing in ICCc neurons. **(A)** Example recording showing adaptation of action potential generation in a tonic/sustained neuron for 20 ms (left) and 200 ms (right) inter-stimulus interval. A single repetition is depicted. Upper trace: membrane potential. Lower trace: graphical depiction of stimulus current. **(B)** Recordings for all ISI tested for the neuron in **(A)**, 10 repetitions each are shown. For clarity only membrane potential traces during the 100 ms stimulus presentation are shown, omitting the interval between the stimuli. **(C,D)** Single example recordings **(C)** and 10 repetitions of all ISI tested **(D)** for a typical phasic/onset neuron, presentation as in **(A,B)**. Note that this neuron does not fire AP for ISI ≤ 5 ms. **(E1–6)** Adaptation functions for the same tonic/sustained neuron as in **(A,B)** for the ISI-dependent ratio of **(E1)** AP count during 100 ms stimulation, **(E2)** amplitude of the first AP during each stimulation, **(E3)** latency of the first AP with respect to each stimulus onset, **(E4)** duration of the first AP during each stimulation, **(E5)** maximal rising slope of the first AP during each stimulation and **(E6)** maximal falling slope of the first AP during each stimulation. White circles/black line shows mean ± SEM of the ratio for each ISI, green line depicts best double-exponential fit. **(F1–6)** Adaptation functions for the same phasic/onset neuron as in **(C,D)**. Presentation as in **(E)**.

**Figure 4:**
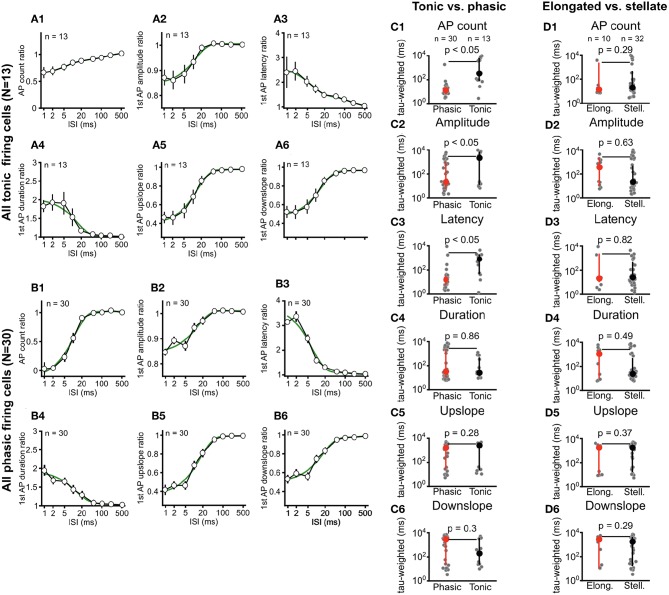
Phasic and tonic ICCc neurons differ in their stimulus-interval dependent adaptation of action potential firing. Dendritic morphology type does not predict adaptation behavior. **(A1–6)** Averaged adaptation function for all (*n* = 13) tonic/sustained neurons. Presentation as in Figure 3E. **(B1–6)** Averaged adaptation function for all (*n* = 30) phasic/onset neurons. Presentation as in Figure 3E. **(C1–6)** Weighted exponential time-constant of the exponential fits (gray dots) of the individual adaptation functions for all phasic (*n* = 30; red marker) and tonic (*n* = 13, black marker) neurons. Bold markers and whiskers depict mean ± SEM. Phasic neurons have significantly more rapid adaptation of AP count, first AP amplitude and first AP latency. **(D1–6)** Weighted exponential time-constant of the exponential fits (gray dots) of the individual adaptation functions for all elongated (*n* = 10; red marker) and stellate (*n* = 32, black marker) neurons. Bold markers and whiskers depict mean ± SEM. No significant differences for any parameter was seen between the morphological groups.

**Figure 5:**
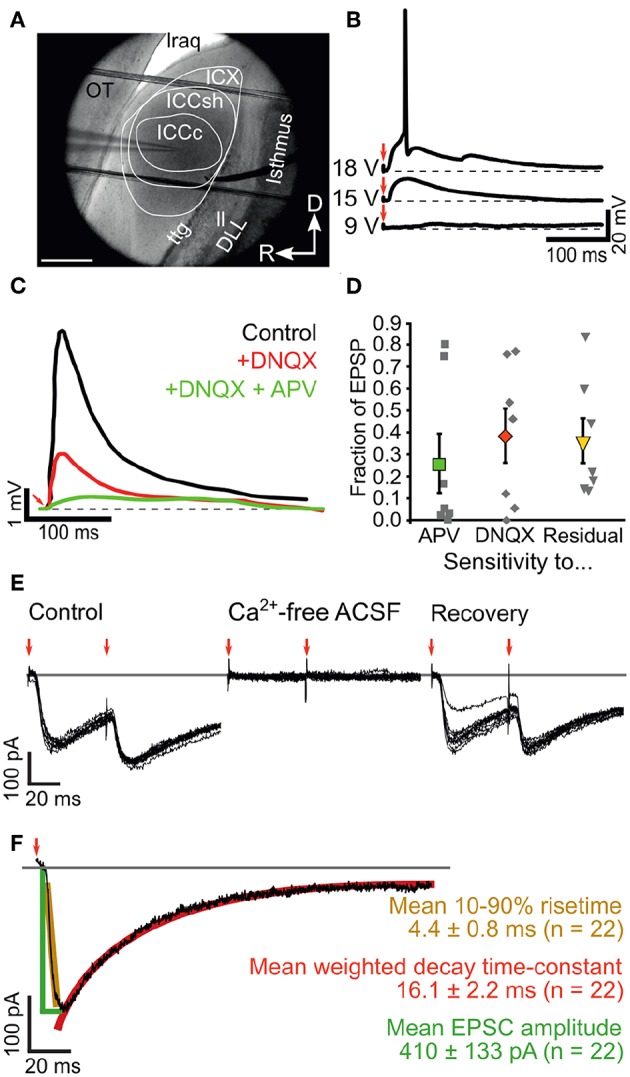
Ascending inputs elicit mixed glutamatergic synaptic events in ICCc neurons. **(A)** IR-DIC image showing the cytochemically defined outlines of the IC subdivisions in acute chicken brain slices as well es placement of patch-electrode (from left) and bipolar stimulation electrode (from right side). OT, optic tectum; lraq, lateral recess of the aqueduct; ttg, tectotegmental tract; ll, lateral lemniscus; DLL, dorsal nucleus of the lateral lemniscus; R, rostral; D, dorsal. **(B)** Example recording traces of EPSP in current clamp mode using perithreshold fiber-stimulation. Red arrows depict stimulation artifact. **(C)** Example of pharmacological identification of glutamatergic receptors underlying the EPSP generation. Black line: control EPSP stimulation, red line: wash-in of the blocker DNQX reveals high contribution of AMPA-type glutamate receptors in this neuron, green line: wash-in of the blockers DNQX + APV reveals contribution of NMDA-type glutamate receptors to the EPSP. Note strongly different kinetics of residual EPSP (green line). **(D)** Fraction of NMDA- (green square) and DNQX-sensitive (red diamond) EPSP amplitude and fraction of residual EPSP amplitude (yellow triangle) for *n* = 7 neurons (gray squares show all data). Bold markers and whiskers depict mean ± SEM. **(E)** Example recording of membrane currents elicited with double lemniscal fiber-stimulation. Red arrows mark stimulation events. Note the paired-pulse depression of the second EPSC and the complete, recoverable abolishment of the membrane currents in calcium-free conditions. **(F)** Analysis of ICCc EPSC amplitude (green lines and text), 10-90% risetime (yellow) and double-exponential decay (red) elicited with single lemniscal fiber stimulation. Group data for *n* = 22 neurons given as mean ± SEM.

If not noted otherwise, non-parametric tests for statistical significance were used throughout this study. Physiological and morphological parameters were compared pairwise between groups of neurons with the Mann-Whitney *U*-Test (Figures 1–4). A Kruskal-Wallis test was used to analyze pharmacological data (Figure 5). In all cases a criterion of *p* < 0.05 for significance was used. Modality of parameter distribution (Figure 1G) was estimated by calculating the bimodality index for finite samples as follows:

(6) $$\begin{array}{l} b=\frac{g^{2}+1}{k+\frac{3{\left(n-1\right)}^{2}}{\left(n-2\right)\left(n-3\right)}} \end{array}$$

with n being the number of observations, g the sample skewness and k the excess kurtosis of the sample. Here, values of $b>\frac{5}{9}$ are usually considered as indicative of bimodality. In addition we estimated the probability density of the sample distribution with an univariate kernel-density estimate (from the python module *statsmodels* 0.8.0) and determined the kernel bandwidth with Silverman's rule of thumb estimator. The statistical independence of shape and firing pattern classification was tested by constructing a contingency-table and performing Pearson's chi-squared test with the Python 2.7 function *scipy.stats.chi2-contingency*. For exponential fits throughout the study we report the goodness-of-fit as the coefficient of determination (*r*^2^) adjusted for degrees of freedom (as returned by the MATLAB function *fit.m*).

### 2.5. Numerical Simulations

For a simple numerical model of ICCc population output we simulated the post-stimulus time histogram (PSTH) of a hypothetical unit receiving inputs from both phasic (70%) and tonic (30%) ICCc units. For this, random spiketimes were generated following either a very flat, shifted and cut-off gaussian distribution (to model tonic neurons) or a steep shifted gaussian distribution (to model phasic neurons). In both cases, the distributions were shifted to simulate an average IC onset latency of 10 ms. Both shifting-value (“latency”) and probability of occurence of a spikes per stimulus (“spike probability”) were dependent on an ISI-parameter according to the average adaptation functions per unit-type we determined experimentally. For this we directly employed the double-exponential fits of AP count and AP latency (Figure 4) per unit type as ISI-dependent modifier functions to the starting target value of spike per stimulus (*n* = 1 for phasic, *n* = 4 for tonic) and onset-latency (10 ms). Please note: only spiketimes were randomly generated, no neuronal or synaptic physiology was simulated. The phasic and tonic simulated PSTH were then summed and normalized for number of repetitions and number of inputs to give instantaneous firing rate of the theoretical target cell in Hz, again without taking any neuronal or synaptic physiology into account. Further, we also normalized to the maximum value to better compare the shape of the resulting PSTH. The maximum rate and the average rate of the last 50 ms were used to calculate onset/sustained-ratio (here, values of 1 would mean a completely flat PSTH and high values indicate a strong emphasize of the phasic onset component). These simulations were performed in MATLAB.

## 3. Results

### 3.1. Morphological Categorization of Chicken ICCc Neurons

In this study we recorded from a total of *N* = 93 neurons in acute slices of the chicken IC. The majority of these neurons (*N* = 69) were confirmed to be located in the ICCc based on *post-hoc* histological analysis (Figures 1A,B). Morphological 3D-reconstruction of dendritic structure was performed in a subset of these identified neurons (*N* = 32), which allowed detailed analysis of dendritic shapes (Figures 1C,D). First, we fitted a minimum volume ellipsoid to both the 3D-data (*N* = 32) and to z-projections of confocal stacks of non-reconstructed ICCc neurons (*N* = 37) and expressed the dendritic shape as either stellate (ratio <2) or elongated (ratio >2) based on the long to short axis ratio, following Niederleitner and Luksch (2012). The mean shape ratio of ICCc neurons was 1.8 ± 0.7 (*N* = 69). Overall, we found 67% of ICCc neurons to be of stellate morphology (46/69 neurons) and 33% (23/69 neurons) to be of elongated morphology. In the subset of neurons in which we performed detailed morphological analysis, 63% (20/32 neurons) were of stellate morphology. ICCc dendritic trees had on average a total pathlength of 1520 ± 745μm (*N* = 32), with a mean of 25.5 ± 16.0 branchpoints (*N* = 32). ICCc dendrites reached on average a maximal distance of 172 ± 53μm (*N* = 32) from the soma. Sholl-analysis (Figures 1E,F) of dendritic complexity revealed a critical range (CR) of 47.5 ± 22.5μm (*N* = 32) at which a critical value (CV) of 14.7 ± 6.7 intersects (*N* = 32) was reached. ICCc dendrites had on average a mean value (MV) of 7.1 ± 3.3 intersects (*N* = 32).

We also analyzed biophysical properties of ICCc neurons by performing current-clamp recordings in whole-cell configuration. Our ICCc neurons had an average resting membrane potential of −53.4 ± 8.5 mV (*N* = 69), average membrane resistance of 302 ± 150*MΩ* (*N* = 69) and average membrane capacitance of 40.5 ± 16.4pF (*N* = 69). The membrane biophysics resulted in an average membrane time-constant of 12.3 ± 7.2 ms (*N* = 69). Action potential threshold current was on average 0.14 ± 0.08 nA (*N* = 69).

We next asked whether the neurons categorized as stellate and elongated represented actual populations of distinct neuronal classes. The distribution of shape ratios (Figure 1G) did not show two completely separate maxima. However, the bimodality index (see Equation 6) of this sample was b = 0.564. Since values of $b>\frac{5}{9}$ (0.555…) are usually considered as indicative of a bimodal distribution we conclude that the distribution of shape ratios agreed better with a mixture of two morphological populations. Additional support for this came from performing a kernel density estimate using a rule-of-thumb bandwidth estimator (resulting bandwidth h = 0.246), which resulted in a kernel-density estimate for the sample of shape ratios more in line with an underlying mixture of gaussians than a single skewed gaussian. Thus, we interpreted the sample of shape ratios found by our morphological analysis to be caused by two distinct populations of neurons as opposed to a wide continuum of shapes resulting from a single diverse neuron class. Accordingly, we divided our biophysical and morphological data into two groups (stellate vs. elongated) and asked, whether the two putative classes of neurons would have an influence on the analyzed parameters. Surprisingly, there were no statistically significant differences in either of the biophysical parameters we analyzed (Figure 1H). Neither resting membrane potential (−53.2 ± 9.3 mV vs. −51.2 ± 9 mV, *p* = 0.39, Figure 1H1), action potential threshold (0.14 ± 0.07 nA vs. 0.13 ± 0.11 nA, *p* = 0.38, Figure 1H2), membrane resistance (325 ± 157*MΩ* vs. 294 ± 174*MΩ*, *p* = 0.2, Figure 1H3), membrane capacitance (44.8 ± 16.5pF vs. 37 ± 16.8pF, *p* = 0.18, Figure 1H4) nor membrane time-constant (13.8 ± 6.6 ms vs. 10.9 ± 7 ms, *p* = 0.09, Figure 1H5) were significantly different for stellate vs. elongated cells.

However, when we compared the morphological parameters between stellate and elongated neurons, statistically significant differences resulted (Figure 1J). We would like to emphasize that the morphological parameters analyzed here and the grouping criterion (i.e., the shape ratio) were derived from independent analyses. Stellate neurons had on average a significantly greater total dendritic path length (1707 ± 774μm vs. 1209 ± 571μm, *p* < 0.05, Figure 1J1) but reached to a shorter average maximal distance from the soma (154 ± 39μm vs. 202 ±60 μm, *p* < 0.05, Figure 1J2). Although the difference in the average number of branch points failed to reach statistical significance (29 ± 18 vs. 19 ± 9, *p* = 0.18, Figure 1J3), the Sholl analysis parameters critical value (17.0 ± 7.0 vs. 10.9 ± 3.6, *p* < 0.01, Figure 1J4) and mean value (8.6 ± 3.4 vs. 4.5 ± 1.2, *p* < 0.001, Figure 1J5) were on average significantly different between stellate and elongated neurons. The critical range, on average, was not statistically different (51 ±23μm vs. 42 ±20μm, *p* = 0.19, Figure 1J6). Taken together, stellate ICCc neurons had significantly more complex dendritic trees that densely covered an area closer to the soma of the neuron. The dendritic trees of elongated neurons on the other hand reached further from the soma of the neuron. Morphological differences likely translate into a different function in information processing in the ICCc for these neurons given the highly laminated organization of this brain area.

### 3.2. Two Physiological Classes of Neurons in the Chicken ICCc *in vitro*

Neuron classes defined by dendritic morphology did not differ in their basic biophysical parameters. In order to investigate whether physiological parameters beyond basic biophysics could be used to characterize these classes of neurons in the chicken ICCc, we analyzed the action potential firing patterns. Indeed, we found that AP firing patterns represented a neuron classification orthogonal to the morphological grouping (Figure 2). AP firing pattern was classified in all *N* = 93 IC neurons. A number of neurons (32%, 29/93) showed tonic firing behavior characterized by ongoing generation of AP during stimulation with suprathreshold depolarizing currents (Figure 2A). From these families of stimulus currents we generated current-voltage curves as shown in Figure 2B. AP frequency in response to various stimulus currents was analyzed by constructing current-frequency curves (Figure 2C). For tonic neurons these were characterized by higher AP frequencies (i.e., multiple AP per stimulus) and monotonically increasing frequencies with increasing current. Phasic neurons (Figures 2D–F), on the other hand, occurred more frequently (68%, 63/93). These neurons were characterized by a single (or two) AP generated only at the onset of the stimulus, almost regardless of stimulus amplitude. This was clearly visible in the saturating shape of the current-frequency plot (Figure 2F).

When we compared biophysical features of ICCc neurons based on their AP firing behavior (Figure 2G), statistically significant differences became evident. While the resting membrane potential was very similar (−54.1 ± 8.7 mV vs. −51.9 ± 8.0 mV, *p* = 0.35, Figure 2G1), AP threshold (0.16 ± 0.09 nA vs. 0.1 ± 0.08nA, *p* < 0.01, Figure 2G2) and membrane resistance (284 ± 151*MΩ* vs. 342 ± 137*MΩ*, *p* < 0.05, Figure 2G3) differed significantly between phasic and tonic groups. Membrane capacitance did not appear to be different (39 ± 18 pF vs. 44 ± 11pF, *p* = 0.1, Figure 2G4), which was in line with the morphological analysis presented in the next paragraph. The membrane time-constant, on the other hand, was significantly different (11.2 ± 7.1 ms vs. 14.8 ± 6.8 ms, *p* < 0.05, Figure 2G5) between phasic and tonic neurons. We thus found that phasic neurons were harder to excite but had more rapid membrane time-constants, while tonic neurons were easier to excite but showed slower membrane time-constants.

Next we asked whether the AP firing behaviors corresponded to the morphological classification. When we compared the average dendritic shape ratio (1.9 ± 0.75 vs. 1.8 ± 0.58, *p* = 0.9, Figure 2G6) between phasic and tonic neurons, no statistical differences were evident. In fact, when we performed Pearson's chi-square test we found that the parameters of shape and of AP firing pattern were statistically independent from each other (χ^2^ = 0.364, degrees-of-freedom = 1, *p* = 0.546). Further, none of the simple or derived morphological parameters (Figures 2H1–6) from the 3D reconstruction of neurons appeared statistically different between phasic and tonic neurons.

We concluded therefore, that the AP firing behavior of the neurons did not predict the dendritic shape or complexity. In other words, both physiological AP firing modes appeared in comparable statistical frequencies in the groups of morphologically defined stellate and elongated chicken ICCc neurons.

### 3.3. Interval-Dependent Dynamics of Intrinsic Neuronal Excitability in the Chicken ICCc *in vitro*

With our biophysical characterization we established that phasic and tonic neurons differed in their excitability. However, we believed that intermittent, repeated activity (possibly at intervals in the range of milliseconds) was the more physiologically relevant stimulus regime for an auditory brainstem neuron. We therefore analyzed the interval-dependency of intrinsic neuronal excitability with double-stimulation in current-clamp mode (Figure 3).

We show here two typical example neurons from our measurement of intrinsic adaptation of excitability for tonic (Figures 3A,B,E) and phasic (Figures 3C,D,F) ICCc neurons in acute slices. In the following we will refer to the AP in response to the first or second stimulus as AP_1_ or AP_2_, respectively, regardless of AP firing behavior. When we stimulated ICCc neurons with pairs of depolarizing currents at short intervals below 20 ms, phasic neurons often failed to generate AP_2_ (Figure 3C). Tonic neurons always generated AP_2_, regardless of the inter-stimulus interval, but the number of generated AP_2_ per stimulus was reduced (Figure 3A). AP_2_ numbers returned to normal at long stimulus intervals (Figures 3A,C). However, the latency and shape of the AP_2_ often still differed for very long ISI (Figures 3B,D). We quantified adaptation for a range of stimulus intervals and repetitions. We not only analyzed AP probability/count but also the latency of the generated AP and kinetic parameters (amplitude, maximum up- and downslopes, AP duration) for every AP. The reason for analyzing AP kinetic parameters was to observe subthreshold adaptative phenomena. By this we mean subtle effects on ion channels directly or indirectly involved in AP generation, which do not overtly change the total number of AP generated but nevertheless might substantially affect the temporal precision and/or time-course of the AP and thus information processing in the ICCc. We expressed the parameters as the ratio of parameters for AP_2_ divided by AP_1_, to highlight the change caused by adaptation. We plotted the obtained ratio as the average of 10 repetitions vs. inter-stimulus interval (ISI) for the tonic (Figures 3E1–6) and phasic (Figures 3F1–6) neurons to obtain adaptation functions for the various parameters. Here the maximal/minimal value is a measure for the severity of adaptative processes. By fitting a double-exponential function to the adaptation functions (green lines in Figures 3E,F) we calculated a weighted time-constant of adaptation for these parameters (as in Equation 3) to also quantify the dynamics of adaptation.

Adaptation of AP number was more severe for the phasic example neuron (Figure 3F1) compared to the tonic example neuron (Figure 3E1), but recovered with a faster weighted time-constant (17.3 ms, *r*^2^ = 0.89 vs. 98.1 ms, *r*^2^ = 0.85). The amplitude of AP_2_ shows almost no adaptation with a ratio of 0.96 for the phasic neuron (Figure 3F2), while the AP_2_ amplitude of the tonic example neurons was marginally more adapted (ratio 0.86). In both cases the unrestrained double-exponential fit described the adaptation function of AP amplitude well (*r*^2^ = 0.99 vs *r*^2^ = 0.98), but resulted in very long weighted time-constants of recovery (2.1s vs. 6.2s). Adaptation of AP_2_ latency showed comparable severity for the phasic (Figure 3F3) and tonic (Figure 3E3) neuron, but again recovered faster for the phasic neuron (15.7 ms, *r*^2^ = 0.99 vs. 989 ms, *r*^2^ = 0.83). In line with the lack of adaptation of AP amplitude, the phasic example neuron also showed relatively little change of AP_2_ duration (1.07), up- (0.84) or downslope (0.87; Figures 3F4–6). These mild adaptive effects recovered with very slow weighted time-constants for the phasic cell (0.2s, 3.2s and 4.6s). The tonic cells showed robust adaptation of AP_2_ duration (1.54, tau_w_ = 31 ms, Figure 3E4), maximal upslope (0.47, tau_w_ = 40 ms, Figure 3E5) and maximal downslope (0.58, tau_w_ = 38 ms, Figure 3E6).

Adaptation differed between phasic and tonic neurons with regard to severity and dynamics of recovery. In order to analyze this, we averaged all adaptation functions from tonic neurons (Figure 4A) and phasic neurons (Figure 4B) and, again, fitted double-exponential functions to the resulting group-adaptation functions. For the adaptation of the number of AP_2_ (Figures 4A1,B1), group data confirms that phasic neurons adapt more severely but recover faster from adaptation (tau_w_ = 10.8 ms, *r*^2^ = 0.99). AP_2_ numbers of tonic neurons adapt less severely but recover markedly slower from adaptation (tau_w_ = 62.7 ms, *r*^2^ = 0.99). The individual weighted time-constants of adaptation of AP count (Figure 4C1) were on average significantly smaller for phasic neurons vs. tonic neurons (14 ms vs. 3.7s; *U*-test *p* < 0.05). Group recovery functions of AP_2_ amplitude (Figures 4A2,B2) were very similar for phasic vs. tonic neurons (13.3 ms, *r*^2^ = 0.95 vs. 12.1 ms, *r*^2^ = 0.98), while the individual weighted time-constants of the recovery functions (Figure 4C2) were significantly different (21 ms vs. 2.1s; *U*-test *p* < 0.05). For the AP_2_ latency (Figures 4A3,B3) the group adaptation functions (17.9 ms, *r*^2^ = 0.97 vs. 62.5 ms, *r*^2^ = 0.99) as well as the individual weighted time-constants (16.5 ms vs. 0.81s; *U*-test *p* < 0.05; Figure 4C3) were different. For the kinetic parameters of AP_2_ no differences between phasic and tonic neurons were evident from neither the group adaptation functions (Figures 4A4–6,B4–6), nor the average individual weighted time-constants (Figures 4C4–6).

Neither the group adaptation functions (data not shown), nor the averaged individual weighted time-constants of recovery from adaptation (Figures 4D1–6) showed any significant differences for stellate vs. elongated ICCc neurons. This further corroborated our finding (see section 3.1) that the morphological classification did not predict physiological characteristics in chicken ICCc neurons but rather represented an orthogonal categorization of neurons.

Taken together we concluded that the interval-dependent adaptation of the number and latency of AP_2_ was significantly different between phasic and tonic firing ICCc neurons. A much more pronounced slow-exponential component (which was evident from the group adaptation functions and the consecutively slower weighted time-constant of recovery) contributed to adaptation in tonic neurons. This slow component appeared, on average, to be mostly absent in phasic firing neurons.

### 3.4. Properties of Ascending Synaptic Connections to Neurons in the Chicken ICCc *in vitro*

Next we wanted to study the properties of ascending connections into the chicken ICCc for two reasons. First, in order to understand the underlying cellular mechanisms that govern adaptive behavior in avian ICCc that was observed *in vivo*, it is vital to understand the properties and dynamics of the synaptic input to the cells under consideration. Synaptic dynamics might in fact be a major contributor to adaptation. The avian ICCc neurons are an ideal testbed for this idea, as these neurons should receive rather uniform, narrow-bandwidth ascending inputs from the auditory brainstem via lateral lemniscus fibers (Puelles et al., 1994; Wang and Karten, 2010). In addition to this, very little quantitative data on synaptic properties in the avian IC has been published before.

Upon brief electrical shocks delivered to the ascending lateral lemniscus fibers (Figure 5A), excitatory postsynaptic potentials (EPSP) could be recorded in ICCc neurons (Figure 5B) that gradually increased in amplitude with increasing stimulation voltage due to recruiting of fibers and eventually elicited AP in the postsynaptic cells (Figure 5B). EPSP in ICCc neurons could be reliably evoked at 34.5 ± 23.3V (*N* = 11) stimulation strength. However, this apparent threshold was likely mostly determined by electrode placement and slice condition and thus does not really carry much biological meaning. We nevertheless determined the threshold for every cell and performed pharmacological experiments at 200% threshold level. The just suprathreshold EPSP had an average amplitude of 5.9 ± 2.4 mV (*N* = 8). Notably, we never observed evoked hyperpolarizing synaptic responses one would expect for inhibitory postsynaptic responses (IPSP) at any stimulation level, although spontaneous IPSP were occasionally observed (not shown). To confirm the pure excitatory nature of the ascending lemniscal inputs we applied blockers of glutamatergic synaptic transmission to the bath solution in a first set of experiments. Upon wash-in of 100 μM DNQX a noticable reduction of EPSP amplitudes compared to control conditions at identical stimulation voltage occurred (Figure 5C). The DNQX-sensitive part of the synaptic events accounted on average for 39 ± 12% (*N* = 7; median: 46.1%) of the EPSP amplitude. Wash-in of 100μM DNQX + 50 μM AP5 further reduced the EPSP amplitude (Figure 5C). Thus, the AP5-sensitive part of the synaptic events accounted on average for another 26% ± 13% (*N* = 7; median: 5.1%) of the EPSP amplitude. In most cells a substantial residual EPSP remained that could not be blocked by both DNQX and AP5. This residual component accounted on average for 36% ± 10% (*N* = 7; median: 21.8%) of the EPSP amplitude. A statistical comparison of these data (Kruskal-Wallis test, χ^2^ = 1.68, *df* = 20, *p* = 0.43) did not reveal any significant differences between the pharmacological groups. The identity of the residual EPSP could not be confirmed in our experiments. Neither a cholinergic blocker (50 μM d-tubocurarin, *N* = 3) nor a mix of glycinergic and gabaergic blockers (1 μM strychnine + 10 μM gabazine + 2 μM CGP-55845, *N* = 2) reduced the amplitudes of lemniscally evoked EPSP in ICCc neurons (data from these experiments not shown). We nevertheless concluded that overall a substantial proportion of the synaptic event elicited by ascending lemniscal fibers in chicken ICCc neurons was mediated by DNQX-sensitive, and thus AMPA- or Kainate-type, glutamatergic receptors.

In a different set of experiments we recorded postsynaptic inward currents from ICCc neurons in voltage-clamp mode (V_*H*_ = 60 mV) upon electrical stimulation of ascending lemniscal fibers (Figures 5E,F). To rule out direct stimulation we showed that the occurrence of these inward currents depended on the presence of calcium ions in the bath solution (Figure 5E). Further indication for synaptic vs. direct origin of these inward currents was provided by the common occurence of short-term plasticity: note the reduction of the inward current amplitude in Figure 5E upon the second pulse (red arrows). We will return to short-term plasticity in section 3.5. We initially analyzed the static properties of lemniscally evoked synaptic inward currents in *N* = 22 ICCc neurons (Figure 5F) at room temperature and 200% threshold level. The inward currents were rapidly-rising (mean 10%-90% risetime of 4.4 ± 0.8 ms, *N* = 22) and quite strong (mean amplitude 410 ± 133pA, *N* = 22). They showed a rapid decay best described by a double-exponential function (mean weighted decay time-constant of 16.1 ± 2.2 ms, *N* = 22).

Taken together we report here for the first time on ascending lemniscal synaptic connections in the chicken ICCc. These lemniscal axons elicited fast and strong inward currents via mostly AMPA-glutamatergic receptors. The kinetics and amplitude of these lemniscal synaptic events were in line with the function of the ICCc in temporal coding and sound-localization.

### 3.5. Synaptic Dynamics in the Chicken ICCc *in vitro* Are Dominated by Short-Term Depression

**Figure 6:**
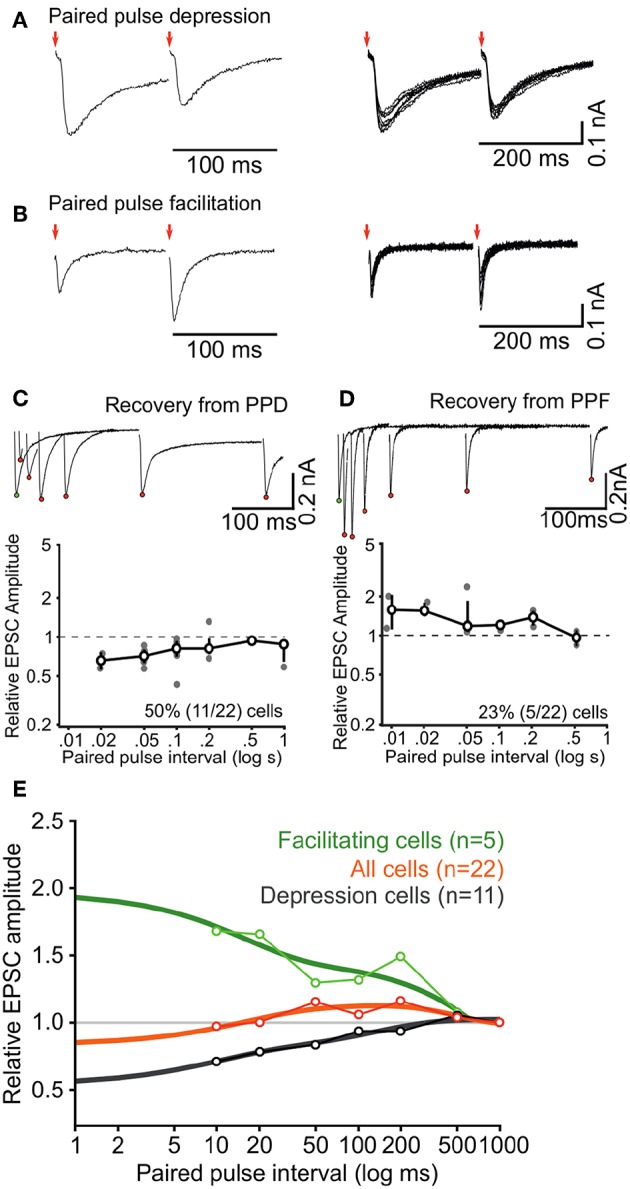
Short-term synaptic dynamics of ascending inputs into ICCc are dominated by short-term depression. **(A,B)** Example paired-pulse EPSC recordings for two different inter-pulse intervals in a depressing neuron **(A)** and a facilitating neuron **(B)**. **(C)** All paired-pulse recordings for the neuron in **(A)** (upper inset, red dots depict EPSC_2_ peak amplitudes, white dot depicts EPSC_1_ peak amplitude) and group averaged recovery from paired-pulse depression for all (*n* = 11) depressing neurons. Bold line, markers, and whiskers: mean ± SEM, gray dots: individual data **(D)** All paired-pulse recordings for the neuron in **(B)** (upper inset) and group averaged recovery from paired-pulse facilitation for all (*n* = 5) facilitating neurons. Presentation as in **(C)**. **(E)** Group averaged recovery from paired-pulse plasticity functions for all neurons (*n* = 22, orange line) and replotted for facilitating neurons (*n* = 5, green) and depressing neurons (*n* = 11, black), fitted with double (facilitation and depression) or triple (all) exponential functions to quantify the time course of short-term plasticity (thick lines).

After having established the static or resting properties of the ascending excitatory connections to ICCc neurons we wanted to explore their dynamics upon repeated stimulation at short intervals. As detailed above synaptic short-term plasticity may be considered a major contributor to adaptive processes in many neuronal system. To our knowledge, nothing is known yet about short-term synaptic plasticity in the chicken ICCc. In order to get a first estimate of short-term synaptic dynamics we recorded in whole-cell voltage clamp mode from *N* = 22 ICCc neurons and employed a paired-pulse stimulus paradigm (Figure 6). We found that the majority of ICCc neurons (50%, 11/22) showed interval-dependent short-term depression (Figure 6A). Surprisingly, other ICCc neurons (23%, 5/22) showed interval-dependent short-term facilitation (Figure 6B) and a number of ICCc neurons (27%, 6/22) showed some a mixture of both depression and facilitation and/or could not be clearly classified as either (not shown). By plotting the normalized average EPSC amplitudes vs. the paired-pulse interval we obtained functions of recovery from short-term plasticity for ICCc neurons. This was illustrated for a neuron with short-term depression in Figure 6C, upper plot. We average all paired-pulse recovery functions for depressing cells (Figure 6C, lower plot). The same analysis was performed for facilitating ICCc neurons (Figure 6D), the unclear/unclassified neurons (not shown) and all neurons (not shown). We then fitted double-exponential functions to the averaged recovery functions of depressing or facilitating ICCc (Figure 6E) and obtained a weighted time-constant of recovery. The depressing ICCc neurons on average recovered from short-term plasticity with τ_*w*_ = 76*ms* (*r*^2^ = 0.87). Facilitating ICCc neurons recovered slower (τ_*w*_ = 338*ms*, *r*^2^ = 0.71). For the average of all ICCc neurons a tri-exponential fit (double-exponential depression + single-exponential facilitation) was sufficient to fit the data (*r*^2^ = 0.75, τ_*fast*−*depr*._ = 14.8 ms, τ_*slow*−*depr*._ = 170 ms, τ_*facil*._ = 194 ms).

Did the categories of short-term plasticity correspond with the physiological groups determined before? We have data on AP firing pattern for all *N* = 22 neurons analyzed in this section. When depressing synaptic inputs were observed, 63% (7/11) of neurons showed tonic AP firing behavior. When one of the other short-term plasticity categories (facilitating and unclear) were observed, only 37% (4/11) of the neurons showed tonic firing behavior. Whether this indicates a predominance of short-term depression in tonic firing neurons is unclear however, because the frequency of tonic neurons is unusually high (50%, 11/22) in the subset of neurons used for analysis of short-term plasticity.

Keeping the quantitative limitations of our dataset in mind we took the observed numbers of the categories and the severity of short-term plasticity into account and concluded for the population of ICCc neurons that the ascending lemniscal fibers on average showed moderate short-term depression at short intervals below 20 ms and weak facilitation for medium intervals (20 ms to 200 ms), before returning to baseline state at intervals of around 500 ms.

### 3.6. Adaptation Changes the Representation of Stimulus Onset in Chicken ICCc Neurons

We described firing pattern specific intrinsic adaptation for ICCc neurons in acute chicken brain slices (see section 3.3). We next wanted to estimate the impact this finding would have on information processing in ICCc neurons *in vivo*. For this, we established a simple first-order numerical simulation that uses the following assumptions based on our *in-vitro* findings:

(1) A hypothetical target neuron of ICCc output (most likely in the shell of the central nucleus of the inferior colliculus) was contacted by a high number of ICCc neurons, with the phasic and tonic neurons distributed according to the frequency of occurrence in our dataset (we chose: 70% phasic, 30% tonic).

(2) At rest (no adaptation) each theoretical ICCc output neuron produced a similar maximal number of AP as the *in-vitro* neurons during 100 ms current stimulation, which we chose to be one AP for phasic and four AP for tonic neurons (cf. Figure 2). In the adapted state the spike probability was reduced according to the adaptation functions of AP count (Figures 4A1,B1).

(3) Onset latency of responses to a given ongoing sound stimulus at rest were identical for the classes of neurons we found *in-vitro* (we chose 10 ms). In the adapted state, onset latency was prolonged according to the adaptation functions of the AP latency for each neuron class (Figures 4A3,B3).

(4) The *in-vitro* firing patterns were translated into a specific probability distribution of spike occurrence relative to stimulus onset, so that phasic neurons had a steep gaussian distribution of spiketimes around the onset of the stimulus (plus latency) and the tonic neurons had a very shallow (almost uniform) gaussian distribution aligned with the total duration of the stimulus that was cut off both at the onset (plus latency) and offset of the stimulus. No neuronal or synaptic physiology was simulated, only the numbers and times of AP occurrence was generated according to the premises outlined above. We admit that this represents a substantial simplification and that the resulting distribution of spiketimes represented only a very rough first-order approximation of onset and sustained response patterns described *in vivo*.

In addition to these simplified assumptions, the first order numerical model also ignored synaptic dynamics.

**Figure 7:**
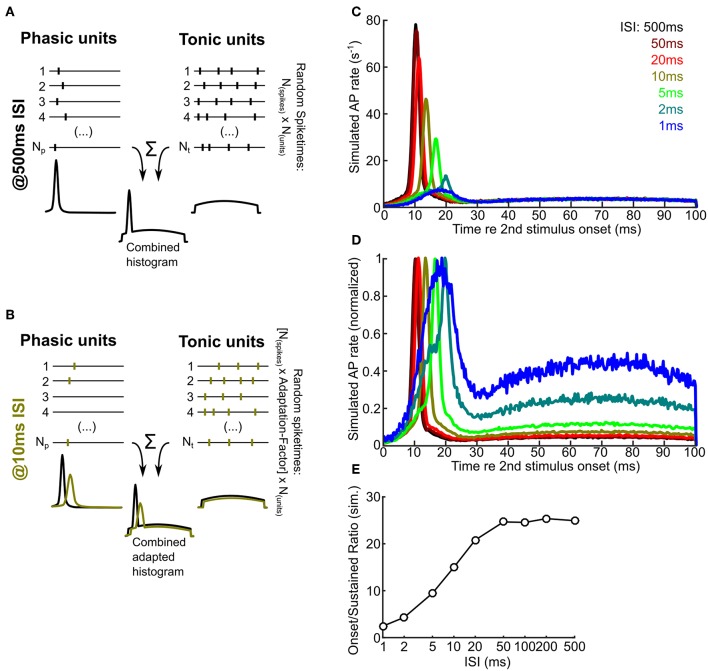
A numerical model predicts that intrinsic adaptation alters the temporal characteristics of ICCc output. **(A,B)** A schematic drawing illustrating the principle of the numerical simulation. Random spiketimes are generated for a large number (N_p_, N_t_) of phasic (**A**,left) and tonic (**A**,right) neurons, shown as black traces (vertical bars depict occurence of simulated action potentials). The peristimulus time histogram of the unit types is shown below the example traces and illustrate the random distributions from which the spiketimes are drawn. All spiketimes of all units (N_p_ + N_t_) are combined to generate a peristimulus time histogram. In the adapted state **(B)**, the spike probability is lower and the onset latency is larger (cf. Figure 4). Green bars / histograms depict the simulated adapted response, black histograms as in A for comparison. Please note that neither cellular nor synaptic mechanisms are simulated. **(C)** Simulated ICCc output as received by a hypothetical ICCsh neuron. Averaged PSTH of the second response for different ISI (from black, 500 ms to blue, 1 ms). Note the strong reduction of the onset peak and the shift of the latency of maximal response. **(D)** Simulated ICCc output as in **(C)** shown as averaged PSTH of the second response for different ISI. Note the drastic increase of the relative amount of AP generated during the ongoing part of the stimulus. Presentation as in **(C)**. **(E)** Quantification of the ratio between maximal AP rate in the onset-peak and the average ongoing AP rate for different ISI.

An schematic overview of the numerical simulation is shown in Figure 7A for the unadapted and in Figure 7B for an adapted state. Note that the AP probability is smaller in the adapted state and the latency of the generated AP is increased. The reduction of the AP probability for phasic or tonic neurons and the amount of latency increase was looked-up in the population adaptation curves in Figures 4A1,A3,B1,B3.

We randomly generated 100,000 ICCc responses to a sound stimulus presented 500 ms after the (unsimulated) first stimulus presentation and plotted a histogram of spiketimes relative to the simulated stimulus onset (ICCc output PSTH; Figure 7C, dark red line). The number of generated responses could be readily interpreted as resulting from 1000 repetitions of 100 ms stimulus presentation and the observation of a target neuron that received a total of 100 ICCc inputs, 70 phasic firing and 30 tonic firing. The resulting PSTH at 500 ms ISI showed a very pronounced peak of spike rate at 10 ms re the stimulus onset, because at very long ISI the temporally much more confined activity of the phasic neurons strongly dominated the simulated ICCc output. Indeed, simulated instantaneous spike rate at the onset peak was about 25x larger than the average sustained rate during the last half of the stimulus. With reduction of ISI the following trends were observed: the instantaneous rate of the onset peak was gradually reduced from 77.9 APs^-1^ at 500 ms ISI to 7.4 APs^-1^ at 1 ms ISI, the maximum response was shifted to longer latencies from 10 ms at 500 ms ISI to 18.5 ms at 1 ms ISI and the onset peak became increasingly broader. At very short intervals below 10 ms the onset-like characteristic of the PSTH was changed to a phasic-tonic or, at maximal adaptation, almost sustained-like response (see Figure 7C). By normalizing the PSTH for every ISI condition we tried to better visualize (Figure 7D) the temporal shift and broadening of the onset peak. In this figure the shape of PSTH at the minimal ISI of 1 ms (light blue) appeared peculiar. However, the shape may be explained by the residual activity of almost fully adapted phasic neurons at the onset and the broad, shallow distribution of spikes resulting from the minority of tonic neurons. We also quantified the ratio of sustained rate of the PSTH to the maximal rate at the onset of the PSTH and plotted this against ISI (Figure 7E). The ratio changed from 24.9 at 500 ms ISI to 2.4 at 1 ms ISI. We interpret this plot as the spike rate adaptation of the hypothetical ICCsh neuron that was caused by the intrinsic adaptation “upstream” in the ICCc neurons. It showed that with shorter ISI the onset feature of a stimulus segment was less represented and the representation of the ongoing features of the sound stimulus was relatively much more dominant in the ICCc output. We therefore conclude from our numerical simulation that intrinsic adaptation has the potential to change and shape the relative representation of different features of a perceived sound stimulus in the avian ICCc.

## 4. Discussion

In this study we showed intrinsic biophysical and synaptic dynamics of neurons in the chicken ICCc that shape adaptation to repeated stimulus presentation at this level of the auditory pathway. We found with whole-cell recordings in acute brain slices that two classes of neurons could be distinguished in the chicken ICCc based on action potential firing pattern: phasic/onset and tonic/sustained firing neurons. We measured intrinsic adaptation of excitability by analyzing the number and kinetics of action potentials upon repeated stimulation and found tonic neurons to have weaker but more prolonged adaptation whereas phasic neurons showed stronger adaptation but also faster recovery from adaptation. We also characterized ascending glutamatergic synaptic inputs into the chicken ICCc for the first time and demonstrated that a majority of synaptic connections showed a form of short term synaptic depression. Together, intrinsic and synaptic dynamics thus must shape the action potential output of chicken ICCc neurons in a celltype specific manner. In a phenomenological model of ICCc population activity we found that the temporal characteristics of ICCc population output were shifted from strongly responding to the onset of stimulations at long inter-stimulation intervals to emphasizing the encoding of ongoing sections of the stimulus in situations of low inter-stimulation intervals or for sustained inputs. These results show that adaptive processes in the IC can be understood by study of the underlying physiological processes that contribute to the dynamics of excitation on the level of individual neurons.

The connectivity and functional physiology of the avian IC was best described in the barn owl, an auditory specialist for hunting in low light conditions using sound localization (Singheiser et al., 2012b). Accordingly, the brain areas used for sound localization circuitry are enlarged in the barn owl brain (Gutiérrez-Ibáñez et al., 2011) and tuning of the neurons to binaural parameters is very distinct and almost stereotypically precise. This is not necessarily the case in the chicken, an auditory generalist. Histologically, the overall structure and subdivisions of the IC are nevertheless also present in the chicken (Puelles et al., 1994; Niederleitner and Luksch, 2012). Thus, we presume that the pathway described in the owl (Singheiser et al., 2012b) from the brainstem to the ICCc, on to the shell of the ICC and subsequently to space-specific neurons in the external nucleus of the IC is also present in the chicken. However, tracing studies showed that ascending binaural inputs from the ITD-coding nucleus laminaris neurons only formed a small subset of inputs in to the chicken IC (Wang and Karten, 2010), a substantial part of the histochemically defined ICCc received inputs from other parts of the cochlear nucleus complex. In accordance with this, a recent physiological study in anesthetized chicken found the same physiological response types known from the barn owl IC, albeit embedded in a greater variety of response types and unit classes (Aralla et al., 2018). The same study suggested that in principle the grid-like organization of tuning to physical parameters (i.e., ITD and frequency) as described in the barn owl (Wagner et al., 2002; Bremen et al., 2007) can be found in the chicken, in a somewhat more diffuse form. Thus, we cannot rule out that some of the diversity of intrinsic and especially synaptic dynamics we observed between unit types in our data can be explained by neurons belonging to completely different ascending streams of information, i.e., the narrow-band ITD coding pathway vs. the frequency and intensity encoding portion of the auditory pathway (Wang and Karten, 2010). It was reported from the mammalian IC that neurons belonging to the non-lemnicscal pathway usually show a greater diversity of adaptive phenomena (Nelken, 2014). Based on our reconstructions we located all neurons included in this study in the ICCc, which we defined immunocytochemically following the work of Puelles et al. (1994) and Niederleitner and Luksch (2012). Lacking conclusive histological evidence we cannot say whether this immunocytochemically defined ICC-subdivision is identical to the area of the ICC receiving ITD-sensitive inputs, as shown by Wang and Karten (2010). We conclude that it appears likely that in the chicken a greater variety of physiological and functional properties can be explained by a greater proportion of the non-ITD coding components of the ascending auditory connections, even in the area identified as the core of the ICC.

In our current study we described physiologically defined groups of neurons in the ICCc of the chicken with regards to action potential firing pattern, biophysical properties of the membrane as well as strength and dynamics of the intrinsic and synaptic adaptation. From our data and other studies it becomes apparent that neurons in the IC do indeed form distinct functional categories best described by a combination of parameters (Ito and Oliver, 2012). For example, we describe phasic firing neurons that not only showed biophysical properties tuned toward temporal precision but also recovered faster from adaptation then other ICCc neurons. One is tempted to speculate that the phasic/onset neurons play a functional role in the ITD-coding narrow-band part of the IC circuitry and are specialized to encode differences in onsets or transients of sounds with high precision. However, our results showed no clear correlation between morphology and physiology or function of the IC neurons. By analyzing the 3D structure of the dendritic trees in detail we could indeed confirm earlier reports from the chicken IC that stellate and elongated neurons represent distinct morphological groups (Niederleitner and Luksch, 2012), possibly related to the laminar functional organization of the ICC (Schreiner and Langner, 1997; Wagner et al., 2002; Malmierca et al., 2008). Interestingly, there is not a single physiological parameter that on average differed between these morphologically defined groups. This is in line with reports from the mammalian IC (Peruzzi et al., 2000). Although a greater diversity of physiologically defined neuron groups were found in the mammalian IC (Peruzzi et al., 2000), possibly due to the postnatal development stage usually used in these experiments, no correlations between form and physiology were apparent in the mammalian IC either. In fact, the understanding of IC circuitry is hampered by the lack of functionally defined neuron types. Only very recently have studies, employing genetic and optogenetic approaches, begun to identify and characterize molecularly defined classes of IC neurons in mice (Goyer et al., 2019). We hope that these studies will finally be able to bridge the gap between *in-vitro* studies and *in-vivo* physiology in the IC and contribute much to resolving the functional circuitry of the IC in both mammals and other vertebrates.

To our knowledge we are the first to characterize ascending synaptic connections into the ICCc of birds. Similar to ascending inputs into the mammalian ICC (Ma et al., 2002; Wu et al., 2002, 2004; Sivaramakrishnan and Oliver, 2006) we describe glutamatergic connections that are mediated to a large degree by both AMPA- and NMDA-type glutamate receptors. This is also in line with the few studies that analyzed synaptic connections between the shell of the ICC and the external nucleus in birds (Penzo and Peña, 2009, 2011). These studies also suggested a combined AMPA- and NMDA-mediated glutamatergic transmission. The postsynaptic currents elicited by lemniscal fiber-stimulation have a much smaller amplitude and much slower kinetics than postsynaptic currents measured in the brainstem nuclei of the auditory pathway (Brenowitz and Trussell, 2001; Goyer et al., 2015), in line with the lesser degree of physiological and morphological specialization. However, compared to reports from the mammalian IC, the ICCc EPSC have similar rise and decay kinetics (Ma et al., 2002; Wu et al., 2002, 2004; Sivaramakrishnan and Oliver, 2006). Furthermore, the EPSC in the ICCc are larger than EPSC recorded in the external nucleus of the IC in the chicken (Penzo and Peña, 2009). We also analyzed short-term plasticity for ascending synaptic connections in the ICCc. The majority of synapses in our dataset showed a form of short-term depression. Compared to synapses in the lower auditory pathway (Cook et al., 2003; Oline and Burger, 2014; Goyer et al., 2015) the short-term depression is not very severe which is surprising given the embryonic recording age of our dataset. We conclude that ascending synaptic connections in the ICCc show a certain resistance against short-term depression. Unfortunately, there is little data on short-term plasticity in the IC to compare our results with. Similar to our findings one study reports the occurrence of both short-term depression (caused mostly by the AMPA component) and facilitation (due to the NMDA component) in glutamatergic synapses in the rat IC (Wu et al., 2004). The relative differences in the abundance of AMPA vs. NMDA channels in ICCc neurons would thus provide an elegant explanation of the diversity of short-term dynamics we found. For technical reasons we however did not systematically check the AMPA vs. NMDA ratio in the neurons for which we quantified short-term plasticity. At the ICCsh to ICX synapse at least two types of long term plasticity have been described (Penzo and Peña, 2009, 2011), but short-term plasticity was not analyzed. Overall we conclude that our dataset is a valuable first approach to describing the synaptic dynamics in the IC, but more studies need to be performed before a definitive answer about the role of synaptic dynamics in sound encoding in the IC can be given.

We sought to better understand adaptive phenomena described in studies performed in anesthetized animals by analyzing intrinsic and synaptic dynamics of the ICCc neurons. But do our results agree with the *in-vivo* studies? First, one has to keep in mind that by studying the processes that contribute to adaptation in isolation, a considerable amount of complexity might be missed. This complexity might arise from dynamic interactions between various processes, which is not possible in the *in-vitro* system employing simple current stimuli. Second, *in vivo* neurons receive a high number of synaptic contacts as inputs, which might fundamentally follow different physiological rules than we observe with current stimuli *in vitro*. Nevertheless, we think that we can compare the adaptation of action potential count and first AP latency to physiological data *in vivo*, albeit with some caution. Furthermore, our recordings were performed at room temperature. Usually a factor of 3 can be assumed for every 10°C difference (Hille, 2001). This means that the single-exponential or fast double-exponential time-constants of the intrinsic parameters we documented (≤ 20 ms) could almost match the very fast components of adaptation (1.5 ms) that were sometimes reported *in vivo* (Singheiser et al., 2012a). Due to the rapid time-constant this type of adaptation of excitability is most likely related to the relative refractory period (Kuenzel et al., 2011; Yang and Xu-Friedman, 2015) and thus mediated by sodium channel inactivation and lingering potassium channel activation. The shape of the responses, especially for the phasic firing neurons, and the responses at the shortest ISIs could also be shaped by low-voltage activated potassium channels of the Kv1 family, which are expressed in the auditory pathway (Trussell, 1997). This could also explain the more severe adaptation at short intervals present in phasic firing ICCc neurons in our study. The majority of *in-vivo* studies in the IC report time-constants of adaptation in the range of tens of milliseconds or longer (Gutfreund and Knudsen, 2006; Singheiser et al., 2012a; Ferger et al., 2018). We conclude that only the synaptic short-term depression we demonstrated and the slower double-exponential changes of excitability can thus fully explain *in-vivo* adaptation phenomena. In the bouton-like glutamatergic synapses of the IC short-term depression is most likely mediated by vesicle pool depletion (Friauf et al., 2015). The slow changes of intrinsic excitability on the other hand could be mediated by calcium-activated potassium channels such as the BK or SK channels that cause repolarization and slow afterhyperpolarization. Indeed, calcium-activated potassium currents (K_*Ca*_) have been reported to occur differentially in physiologically defined neuron types in the mouse IC (Sivaramakrishnan and Oliver, 2001). These authors find that onset type neurons lack K_*Ca*_ while many sustained firing neurons show various types of K_*Ca*_ currents. This would provide an explanation for smaller contribution of slow adaptation components in the chicken ICCc phasic neurons, but K_*Ca*_ was not investigated specifically in our study.

In this study embryonic brains were examined. Although the structures projecting to the ICCc in the auditory brainstem are quite mature already before hatching (Gao and Lu, 2008), further maturation of ICCc physiology and circuitry is likely to be expected. Chickens can indeed hear airborne sounds as early as P17 (Jones et al., 2006) and show sensory functionality immediately after hatching (only one or two days after the time period examined in our study). In mammals, the inferior colliculus is known to develop toward mature-like functions for several days after the onset of hearing (Shnerson and Willott, 1979). Also, the physiology of ascending synaptic inputs still changes in the first one or 2 weeks after hearing onset (Kitagawa and Sakaba, 2019). Very little information is unfortunately available about maturation of IC physiology in the chicken. We conclude that the chicken ICCc is functional but not yet fully matured at the embryonic ages we examined. We thus caution the reader that the absolute values of time-constants of adaptation we describe probably will differ between the late embryonic and the mature state.

With the help of a phenomenological model we predicted drastic changes of the shape of the response to sound stimuli in subsequent neurons of the IC (i.e., the shell of the ICC), caused by the differential adaptation in ICCc neurons. We supposed that subsequent neurons receive an bouquet of input types simply based on the distribution of neurons types we found. The number of ICCc axons converging on ICCsh neurons and their physiological identity has not been documented. Also, no neuronal or synaptic physiology whatsoever was included in the model. Thus, some assumptions of our model are highly speculative. However, the fact that the shape of the PSTH is altered by adaptive processes has been described before in other animals or brain areas (Epping, 1990; Pérez-González and Malmierca, 2012). This challenges the classical view that the shape of the PSTH conveys the fixed coding or information processing function of the neuron. Our model rather suggests that due to the adaptation of their inputs alone ICCsh neurons respond maximally to different aspects of the sound stimulus, dependent on the auditory context. In long intervals (or silence) onsets and transients cause strong activation of the majority of (phasic) ICCc neurons. This would facilitate detection and localization of sudden stimuli in quiet surroundings (cf. Dean et al., 2005). During ongoing stimulation the onset neurons strongly adapt and the information is then mainly processed by sustained neurons, which would possibly emphasize identification and analysis of sounds by spectral and intensity cues. We hypothesize that due to differential adaptation of their inputs the neurons in the IC circuitry could thus participate in several quite different physiological roles depending on stimulus context. Adaptative processes are indeed often thought to underlie even more advanced auditory processing functions like novelty detection (Ulanovsky et al., 2003), contrast enhancement (Willmore et al., 2016; Cooke et al., 2018), auditory attention (Fritz et al., 2007) or segregation of auditory streams (Scholes et al., 2015). Whether the dynamic change of stimulus preference of ICCsh neurons through adaptation can be demonstrated in anesthetized animals must be subject to further studies.

## Data Availability

The datasets generated for this study are available on request to the corresponding author.

## Ethics Statement

All experimental procedures performed on animals in this study were approved by the local animal welfare officer and state authorities (Landespräsidum für Natur, Umwelt und Verbraucherschutz Nordrhein-Westfalen, Recklinghausen, Germany).

## Author Contributions

TK conceived and designed the study. SM, JW, and TK performed experiments and analyzed data. SM and TK wrote the manuscript. All authors discussed results and commented on the manuscript.

### Conflict of Interest Statement

The authors declare that the research was conducted in the absence of any commercial or financial relationships that could be construed as a potential conflict of interest.
